# Higher order solitary solutions to the meta-model of diffusively coupled Lotka–Volterra systems

**DOI:** 10.1186/s13662-021-03300-4

**Published:** 2021-02-25

**Authors:** Inga Timofejeva, Tadas Telksnys, Zenonas Navickas, Romas Marcinkevicius, Minvydas Ragulskis

**Affiliations:** 1 Center for Nonlinear Systems, Kaunas University of Technology, Studentu 50-147, Kaunas, LT-51368 Lithuania; 2 Department of Software Engineering, Kaunas University of Technology, Studentu 50-415, Kaunas, LT-51368 Lithuania

**Keywords:** 35C08, 34A25, 34D06, Nonlinear differential equation, Analytical solution, COVID model

## Abstract

A meta-model of diffusively coupled Lotka–Volterra systems used to model various biomedical phenomena is considered in this paper. Necessary and sufficient conditions for the existence of *n*th order solitary solutions are derived via a modified inverse balancing technique. It is shown that as the highest possible solitary solution order *n* is increased, the number of nonzero solution parameter values remains constant for solitary solutions of order $n>3$. Analytical and computational experiments are used to illustrate the obtained results.

## Introduction

Even though solitons (also called solitary solutions) have been first discovered in the 19th century by John Scott Russell [32], later formalized by Korteweg and de Vries [14] and made famous in the mid-20th century by the Fermi–Pasta–Ulam computational experiment [12] and later works by Norman and Zabusky [41], they remain at the forefront of research to this day due to their unique physical properties.

In recent years, the emergence of powerful computer algebra software coupled with a marked rise in computing power has sparked a new interest in the subject. Analytical investigation is central to the construction of solitary solutions and the availability of aforementioned tools has greatly increased the number of studies in this area.

Classical methods used to construct solitary solutions to differential equations include the inverse scattering transform [2], the Bäcklund transform [31] and the Darboux transform [30] methods.

More recently developed techniques that make use of computer algebra software include the simplest equation method and its extensions [15, 17], the equivalent $(G'/G)$-extension and tanh-extension methods [16, 40], and the homotopy analysis method [1]. A novel adaptation of the $(G'/G)$-expansion technique is used to construct solitary wave solutions to the $(2+1)$ dimensional Konopelchenko–Dubrovsky and Kadomtsev–Petviashvili equations in [5]. The $(G'/G)$-expansion method is further adapted and applied to obtain solitary wave solutions to the $(2+1)$-dimensional time-fractional Schrödinger equation and the space-time nonlinear conformable fractional Bogoyavlenskii equations in [4]. A technique based on Lyapunov’s second method is used to conduct an investigation of integrability of Volterra integro-differential systems in [37].

The determination of solitary solutions to differential equations is an important question in applied research. Some recent examples are given below. Soliton crystals have been observed and characterized in monolothic Kerr resonators, which offers a novel way to increase the efficiency of Kerr combs [9]. A generalized hydrodynamics theory based on soliton solutions is developed in [10] and is applied to the Lieb–Liniger model realized in cold-atom experiments. Solitons have been observed in the cell movement of a cellular slime mould in [13]. Biological population models have been shown to possess solitary solutions on multiple occasions, including [3, 29].

The motivation for this study and its contributions to the theory of solitary solutions is given in the next section.

## Motivation

### The diffusive and the multiplicative coupling

Mathematical modeling of interacting dynamical systems is a classical field of research. For example, a diffusive coupling between two (or more) dynamical systems is used to model the effect of synchronization. The paradigmatic model of two diffusively coupled dynamics is described by:

1 $$\begin{aligned} &x'_{t} = F(x) + \gamma (y-x), \end{aligned}$$

2 $$\begin{aligned} &y'_{t} = F(y) + \gamma (x-y), \end{aligned}$$

where *F* is the vector field modeling the isolated chaotic dynamics; *t* is time; *γ* is the diffusive coupling parameter (usually set a positive constant). The systems are said to be completely synchronized when there is a set of initial conditions so that the systems eventually evolve identically in time and the divergence of trajectories of interacting systems is suppressed by the diffusive coupling [27].

However, dynamical systems can be coupled not only with terms representing the diffusive coupling. Another type of the coupling is the multiplicative coupling. A paradigmatic example of such type of coupling is the Lotka–Volterra model:

3 $$\begin{aligned} &x'_{t} = \alpha x - \beta xy, \end{aligned}$$

4 $$\begin{aligned} &y'_{t} = \delta xy - \gamma y, \end{aligned}$$

where *α*, *β*, *γ*, *δ* are positive real constants. Complete synchronization is not possible in the Lotka–Volterra model (except for two trivial equilibriums when both competing species die out or coexist at fixed population levels).

The classical Lotka–Volterra model (3)–(4) reduces to a system of linear uncoupled ordinary differential equations when the coupling constants *β* and *δ* vanish to zero. A more complex variant of the Lotka–Volterra model is the competitive Lotka–Volterra model which is based on the nonlinear logistic equation [20, 38] instead, namely

5 $$ x'_{t} = r x \biggl(1 - \frac{x}{K} \biggr), $$

where *r* is inherent per-capita growth rate, and *K* is the carrying capacity. The competitive Lotka–Volterra model reads [6]:

6 $$\begin{aligned} &x'_{t} = r_{x} x \biggl(1 - \biggl( \frac{x + \beta _{xy} y}{K_{x}} \biggr) \biggr), \end{aligned}$$

7 $$\begin{aligned} &y'_{t} = r_{y} y \biggl(1 - \biggl( \frac{y + \beta _{yx} x}{K_{y}} \biggr) \biggr), \end{aligned}$$

where the parameter $\beta _{xy}$ represents the effect species y has on the population of species *x*, and $\beta _{yx}$ represents the effect species *x* has on the population of species *y*. Both parameters $\beta _{xy}$ and $\beta _{yx}$ are usually set as positive constants due to harmful (competitive) interaction between species. Indeed, the dynamics of the competitive Lotka–Volterra model is more complex compared to the classical Lotka–Volterra model [7].

The competitive Lotka–Volterra model reduces to two uncoupled nonlinear logistic equations when the coupling constants $\beta _{yx}$ and $\beta _{xy}$ vanish. Note that the nonlinear logistic equation is a partial case of the paradigmatic Riccati equation with constant coefficients [28]:

8 $$ x'_{t} = a_{0} + a_{1} x + a_{2} x^{2}, $$

where $a_{0}, a_{1}, a_{2} \in \mathbb{R}$; $a_{2} \neq 0$.

The analogy between the Lotka–Volterra model (3)–(4) and the competitive Lotka–Volterra model (6)–(7) suggests the following model with the multiplicative coupling:

9 $$\begin{aligned} &x'_{t} = a_{0x} + a_{1x} x + a_{2x} x^{2} + \beta _{xy} xy, \end{aligned}$$

10 $$\begin{aligned} &y'_{t} = b_{0y} + b_{1y} x + b_{2y} x^{2} + \beta _{yx} xy, \end{aligned}$$

where $a_{2x},b_{2y} \neq 0$. The system (9)–(10) reduces to two uncoupled Riccati equations with constant coefficients when the coupling coefficients $\beta _{xy}$ and $\beta _{yx}$ vanish. It appears that such models are widely used to describe the interaction between healthy and cancer cells in phenomenological mathematical models of a single cancer tumor [19]. Such models comprising two Riccati-type equations coupled with multiplicative terms are used for the description of prostate cancer treatment with androgen deprivation therapy [42], cancer stem-cell-targeted immunotherapy [35], the maximization of viability time in general cancer therapy [8]. Elliptic and hyperbolic problems that stem from mechanical models also involve Riccati-type equations, as given in the following examples. A Riccati-type hyperbolic boundary value problem is considered in [18]. An elliptic problem related to membrane equilibrium equations is studied in [39].

### The meta-model of coupled prey–predator systems

The mathematical meta-model of diffusively coupled Lotka–Volterra systems on heterogenous graphs in presented in [21]. When the number of systems is limited to two, the model of diffusively coupled predator–prey systems reads [21]:

11 $$\begin{aligned} &x_{1}' = a_{11} x_{1} - \lambda _{1} x_{1} y_{1}, \end{aligned}$$

12 $$\begin{aligned} &y_{1}' = b_{11} y_{1} - \mu _{1} x_{1} y_{1} + \gamma _{1} (y_{2} - y_{1}), \end{aligned}$$

13 $$\begin{aligned} &x_{2}' = a_{12} x_{2} - \lambda _{2} x_{2} y_{2}, \end{aligned}$$

14 $$\begin{aligned} &y_{2}' = b_{12} y_{2} - \mu _{2} x_{2} y_{2} + \gamma _{2} (y_{1} - y_{2}). \end{aligned}$$

The system of diffusively coupled Lotka–Volterra models (11)–(14) reduces into a system of linear coupled differential equations when the multiplicative coupling constants $\lambda _{1}$, $\mu _{1}$, $\lambda _{2}$, $\mu _{2}$ vanish. A natural extension of (11)–(14) is based on the expansion of the basic Lotka–Volterra model by the nonlinear terms (in accordance to (6)–(7)):

15 $$\begin{aligned} &x_{1}' = a_{01} + a_{11} x_{1} + a_{21} x_{1}^{2} + \lambda _{1} x_{1} y_{1}, \end{aligned}$$

16 $$\begin{aligned} &y_{1}' = b_{01} + b_{11} y_{1} + b_{21} y_{1}^{2} + \mu _{1} x_{1} y_{1} + \gamma _{1} (y_{2} - y_{1} ), \end{aligned}$$

17 $$\begin{aligned} &x_{2}' = a_{02} + a_{12} x_{2} + a_{22} x_{2}^{2} + \lambda _{2} x_{2} y_{2}, \end{aligned}$$

18 $$\begin{aligned} &y_{2}' = b_{02} + b_{12} y_{2} + b_{22} y_{2}^{2} + \mu _{2} x_{2} y_{2} + \gamma _{2} (y_{1} - y_{2} ). \end{aligned}$$

System (15)–(18) does represent a meta-model of two diffusively coupled Riccati systems (each system comprises two Riccati equations coupled with multiplicative terms). System (15)–(18) splits into two uncoupled systems described by (9)–(10) when the diffusive coupling constants $\gamma _{1}$ and $\gamma _{2}$ vanish. Analogously, system (15)–(18) splits into four uncoupled Riccati equations (8) when both the diffusive and the multiplicative coupling constants vanish. In other words, the model described by (15)–(18) generalizes the competitive Lotka–Volterra model in the spatial domain.

### The motivation of this paper

The existence of the first-order soliton-type solutions (kink solitons) to Riccati equation (8) is known for decades [28]. Necessary and sufficient conditions for the existence of second-order soliton-type solutions (dark/bright solitons) to system (9)–(10) has been recently reported in [23]. The existence of *n*th order soliton-type solutions to the meta-model of coupled Riccati equations (15)–(18) poses a serious challenge from the mathematical point of view. Providing two answers to the following questions – what is the maximal order *n*, and what are the necessary and sufficient conditions for the existence of solitons up to the *n*th order – is the main objective of this paper.

## Preliminaries

### Definition of the solitary solution

Solitary solutions of the following form [22, 33] are considered in this paper:

19 $$ x(t) = \sigma \frac{\prod_{k=1}^{n}{(\exp (\eta (t-t_{0}))-x_{k})}}{\prod_{k=1}^{n}{(\exp (\eta (t-t_{0}))-t_{k})}}, $$

where $n\in \mathbb{N}$ is the order of the solitary solution, $t_{0},\sigma ,\eta \in \mathbb{R}$, $x_{k},t_{k}\in \mathbb{C}$.

The following independent variable transformation is introduced:

20 $$ \widehat{t} = \exp \bigl(\eta (t - t_{0})\bigr). $$

Using (20) on (19) simplifies the analytical expression of the solitary solution (19) as follows:

21 $$ x(t) = x \biggl(\frac{\ln {\widehat{t}}}{\eta } + t_{0} \biggr)= \widehat{x} (\widehat{t} ) = \widehat{x}= \sigma \frac{X (\widehat{t} )}{T (\widehat{t} )}, $$

where

22 $$ X(\theta )=\prod_{k=1}^{n} ( \theta - x_{k} ), \qquad T( \theta )=\prod _{k=1}^{n} (\theta - t_{k} ). $$

### Solitary solutions to Riccati equations

#### Uncoupled Riccati equations

Consider the following Riccati equation with respect to $x = x(t)$:

23 $$ x' = c_{0} + c_{1} x + c_{2} x^{2}, $$

where $c_{0}, c_{1}, c_{2} \in \mathbb{C}$.

Equation (23) can be transformed via the substitution (20), where $\eta ^{2} = c_{1}^{2} - 4c_{0} c_{2}$ [28], as follows:

24 $$ \eta \widehat{t} \widehat{x}'_{\widehat{t}} = c_{0} + c_{1} \widehat{x}+ c_{2} \widehat{x}^{2}. $$

The solution to (24) reads [28]:

25 $$ \widehat{x}= \sigma \frac{\widehat{t} - s x_{0}}{\widehat{t} - s t_{0}} = \sigma \frac{\widehat{t} - \frac{x_{0}}{t_{0}}\mbox{\ae }_{0}}{\widehat{t} - \mbox{\ae }_{0}}, $$

where $s \in \mathbb{R}$ is a free constant, $\mbox{\ae }_{0} = s t_{0}$ and parameters *σ*, $x_{0}$, $t_{0}$ satisfy the following identities:

26 $$\begin{aligned} &c_{0} = \frac{\sigma x_{0} \eta }{x_{0} - t_{0}}, \end{aligned}$$

27 $$\begin{aligned} &c_{1} = \frac{ (t_{0} + x_{0} ) \eta }{t_{0} - x_{0}}, \end{aligned}$$

28 $$\begin{aligned} &c_{2} = \frac{t_{0} \eta }{\sigma (x_{0}-t_{0} )}. \end{aligned}$$

Thus the solution to (23) is

29 $$ x = \sigma \frac{\exp (\eta t ) - s x_{0}}{\exp (\eta t ) - s t_{0}}. $$

This solution is known as the kink solitary solution [34]. It describes the transition of a system from one steady state to another via a monotonous trajectory.

##### Example

Suppose that the following Ricatti differential equation with respect to $x = x(t)$ is given:

30 $$ x' = -1 + 5 x - 4 x^{2}. $$

**Figure 1:**
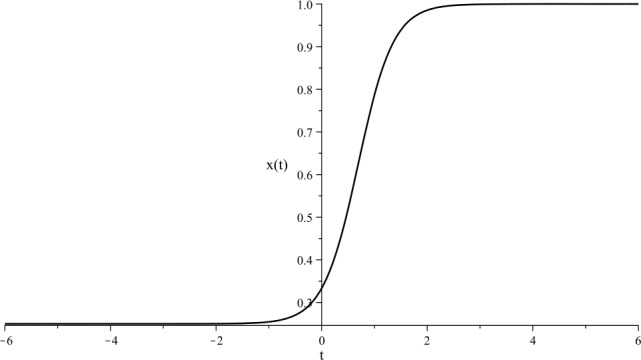
Kink solitary solution to (30) with initial condition $x(0)=\frac{1}{3}$

The kink solitary solution to (30) is displayed in Fig. 1.

#### System of Riccati equations coupled via multiplicative terms

Let us consider the following system of Riccati equations coupled via multiplicative terms:

31 $$\begin{aligned} &x' = a_{0} + a_{1} x + a_{2} x^{2} + a_{3} x y, \end{aligned}$$

32 $$\begin{aligned} &y' = b_{0} + b_{1} y + b_{2} y^{2} + b_{3} x y. \end{aligned}$$

It is shown in [26] that equations (31)–(32) can be uncoupled under the assumption that solutions *x*, *y* are in an inverse relationship:

33 $$ x y = \Omega , \quad \Omega \in \mathbb{R}. $$

In this case, the solutions have the same kink solitary solution form as described in the previous subsection.

Furthermore, it has been shown in [23] that if the condition (33) does not hold, the system (31)–(32) admits the following dark/bright solitary solutions:

34 $$\begin{aligned} &x = \sigma \frac{ (\exp (\eta (t-t_{0}) )-\overline{x}_{1} ) (\exp (\eta (t-t_{0}) )-\overline{x}_{2} )}{ (\exp (\eta (t-t_{0}) )-\overline{t}_{1} ) (\exp (\eta (t-t_{0}) )-\overline{t}_{2} )}, \end{aligned}$$

35 $$\begin{aligned} &y = \gamma \frac{ (\exp (\eta (t-t_{0}) )-\overline{y}_{1} ) (\exp (\eta (t-t_{0}) )-\overline{y}_{2} )}{ (\exp (\eta (t-t_{0}) )-\overline{t}_{1} ) (\exp (\eta (t-t_{0}) )-\overline{t}_{2} )}, \end{aligned}$$

where *σ*, *γ*, *η*, $t_{0}$ are constants and parameters $\overline{t}_{1}$, $\overline{t}_{2}$, $\overline{x}_{1}$, $\overline{x}_{2}$, $\overline{y}_{1}$, $\overline{y}_{2}$ are functions of initial conditions posed for system (31)–(32) at the point $t = t_{0}$.

It is proven in [23] that the above solution holds if and only if the solution parameters satisfy the relations:

36 $$ \frac{ (\overline{x}_{1}-\overline{t}_{1} ) (\overline{x}_{1}-\overline{t}_{2} )}{ (\overline{x}_{2}-\overline{t}_{1} ) (\overline{x}_{2}-\overline{t}_{2} )} = -\frac{\overline{x}_{1}}{\overline{x}_{2}}, \qquad \frac{ (\overline{y}_{1}-\overline{t}_{1} ) (\overline{y}_{1}-\overline{t}_{2} )}{ (\overline{y}_{2}-\overline{t}_{1} ) (\overline{y}_{2}-\overline{t}_{2} )} = -\frac{\overline{y}_{1}}{\overline{y}_{2}}. $$

The system parameters $a_{k}$, $b_{k}$, $k = 0,\dots ,3$ must satisfy the following conditions:

37 $$\begin{aligned} &a_{3} = b_{2}, \qquad a_{2} = b_{3}, \end{aligned}$$

38 $$\begin{aligned} &9a_{0} a_{1} a_{2} + 9b_{0} b_{1} b_{2} - 18a_{0} a_{2} b_{1} - 18 b_{0} b_{2} a_{1} + 3a_{1} b_{1}^{2} + 3b_{1} a_{1}^{2} - 2a_{1}^{3} - 2b_{1}^{3} = 0. \end{aligned}$$

Furthermore, the parameter *η* is

39 $$ \eta = \frac{a_{1}^{2} - a_{1} b_{1} + b_{1}^{2}}{3} - a_{0} a_{2} - b_{0} b_{2}. $$

##### Example

Suppose that the following system of Riccati equations with respect to $x = x(t)$ and $y = y(t)$ is given:

40 $$\begin{aligned} &x' = \frac{136}{11} - \frac{828}{319} x + \frac{29}{187} x^{2} - \frac{550}{1479} x y, \end{aligned}$$

41 $$\begin{aligned} &y' = -\frac{51}{29} + \frac{345}{319} y - \frac{550}{1479} y^{2} + \frac{29}{187} x y. \end{aligned}$$

**Figure 2:**
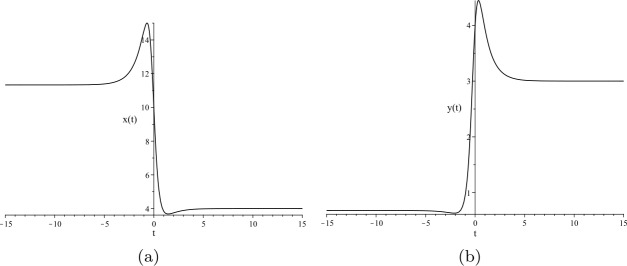
Dark/bright solitary solutions to (40)–(41) with initial conditions $x(0)=10$ and $y(0)=4$: (**a**) $x(t)$ and (**b**) $y(t)$

The dark/bright solitary solutions to (40)–(41) are illustrated in Fig. 2.

It will be demonstrated in this paper that the system (15)–(18) also has both kink and dark/bright solitary solutions, as well as admits higher-order solitary solutions.

### Inverse balancing technique

The inverse balancing technique [24] is used in order to obtain necessary and sufficient conditions for the existence of solitary solutions to a system of differential equations, as well as to determine the possible order of such solutions. The main idea of this technique is to insert the solitary solution as an anzatz into the considered model, which yields a system of linear equations from which the model parameters can be determined in terms of solitary solution parameters. The inverse balancing technique is applied to the system (15)–(18) in Sects. 4 and 5.

Note that a direct balancing approach consisting of inserting the solutions of the form (19) into the system (15)–(18) would result in a high-order nonlinear system of algebraic equations with respect to the solution parameters. Direct construction of a solution to this system would not be possible. Due to this, the inverse balancing technique is applied for the analysis of the system (15)–(18).

## Derivation of necessary and sufficient conditions for the existence of solitary solutions to (15)–(18)

The system of equations (15)–(18) can be transformed via the substitution (20) as follows:

42 $$\begin{aligned} &\eta \widehat{t} \widehat{x_{1}}'_{\widehat{t}} = a_{01} + a_{11} \widehat{x_{1}} + a_{21} \widehat{x_{1}}^{2} + \lambda _{1} \widehat{x_{1}} \widehat{y_{1}}, \end{aligned}$$

43 $$\begin{aligned} &\eta \widehat{t} \widehat{y_{1}}'_{\widehat{t}} = b_{01} + b_{11} \widehat{y_{1}} + b_{21} \widehat{y_{1}}^{2} + \mu _{1} \widehat{x_{1}} \widehat{y_{1}} + \gamma _{1} ( \widehat{y_{2}} - \widehat{y_{1}} ), \end{aligned}$$

44 $$\begin{aligned} &\eta \widehat{t} \widehat{x_{2}}'_{\widehat{t}} = a_{02} + a_{12} \widehat{x_{2}} + a_{22} \widehat{x_{2}}^{2} + \lambda _{2} \widehat{x_{2}} \widehat{y_{2}}, \end{aligned}$$

45 $$\begin{aligned} &\eta \widehat{t} \widehat{y_{2}}'_{\widehat{t}} = b_{02} + b_{12} \widehat{y_{2}} + b_{22} \widehat{y_{2}}^{2} + \mu _{2} \widehat{x_{2}} \widehat{y_{2}} + \gamma _{2} (\widehat{y_{1}} - \widehat{y_{2}} ). \end{aligned}$$

Let

46 $$\begin{aligned} &x_{l} (t ) = \widehat{x_{l}} (\widehat{t} ) = \sigma _{1l} \frac{X_{l}}{T}, \qquad y_{l} (t ) = \widehat{y_{l}} (\widehat{t} ) = \sigma _{2l} \frac{Y_{l}}{T}, \end{aligned}$$

47 $$\begin{aligned} &X_{l} = X_{l} (\widehat{t} ) = (\widehat{t} - x_{1l} ) (\widehat{t} - x_{2l} ) \cdots (\widehat{t} - x_{nl} ) = \widehat{t}^{n} + \chi _{(n-1)l} \widehat{t}^{n-1} + \cdots + \chi _{0l}, \end{aligned}$$

48 $$\begin{aligned} &Y_{l} = Y_{l} (\widehat{t} ) = (\widehat{t} - y_{1l} ) (\widehat{t} - y_{2l} ) \cdots (\widehat{t} - y_{nl} ) = \widehat{t}^{n} + \theta _{(n-1)l} \widehat{t}^{n-1} + \cdots + \theta _{0l}, \end{aligned}$$

49 $$\begin{aligned} &T = T (\widehat{t} ) = (\widehat{t} - t_{1} ) (\widehat{t} - t_{2} ) \cdots (\widehat{t} - t_{n} ) = \widehat{t}^{n} + \mbox{\ae }_{n-1} \widehat{t}^{n-1} + \cdots + \mbox{\ae }_{0}, \end{aligned}$$

50 $$\begin{aligned} &X_{l}' = \bigl(X_{l} (\widehat{t} ) \bigr)'_{ \widehat{t}}, \qquad Y_{l}' = \bigl(Y_{l} (\widehat{t} ) \bigr)'_{\widehat{t}}, \qquad T' = \bigl(T (\widehat{t} ) \bigr)'_{\widehat{t}}, \end{aligned}$$

where $\mbox{\ae }_{k}, \chi _{kl}, \theta _{kl} \in \mathbb{C}$, $k = 1,\dots ,n-1$, $l = 1,2$. Note that in this paper the order of the solitary solution is defined by the value of the parameter *n*.

Necessary and sufficient conditions for the existence of solitary solutions to (15)–(18) are further obtained by inserting the solitary solutions (46) into (42)–(45). The system can then be rewritten in the following way:

51 $$\begin{aligned} & \eta \widehat{t} \sigma _{1l} \frac{X_{l}' T - X_{l} T'}{T^{2}} = a_{0l} + a_{1l} \sigma _{1l} \frac{X_{l}}{T} + a_{2l} \sigma _{1l}^{2} \frac{X_{l}^{2}}{T^{2}} + \lambda _{l} \sigma _{1l} \sigma _{2l} \frac{X_{l} Y_{l}}{T^{2}}, \end{aligned}$$

52 $$\begin{aligned} &\eta \widehat{t} \sigma _{2l} \frac{Y_{l}' T - Y_{l} T'}{T^{2}} = b_{0l} + c_{l} \sigma _{2l} \frac{Y_{l}}{T} + b_{2l} \sigma _{2l}^{2} \frac{Y_{l}^{2}}{T^{2}} + \mu _{l} \sigma _{1l} \sigma _{2l} \frac{X_{l} Y_{l}}{T^{2}} + \gamma _{l} \sigma _{2r} \frac{Y_{r}}{T}, \end{aligned}$$

where $c_{l} = b_{1l}-\gamma _{l}$, $l,r = 1,2$, $r \neq l$.

Multiplying both sides of the equations (51)–(52) by $\frac{T^{2}}{X_{l}}$ and *T*, respectively, and rearranging the resulting equations yields:

53 $$\begin{aligned} & \frac{\eta \widehat{t} \sigma _{1l} X_{l}' T}{X_{l}} - \frac{a_{0l} T^{2}}{X_{l}} = \eta \widehat{t} \sigma _{1l} T' + a_{1l} \sigma _{1l} T + a_{2l} \sigma _{1l}^{2} X_{l} + \lambda _{l} \sigma _{1l} \sigma _{2l} Y_{l}, \end{aligned}$$

54 $$\begin{aligned} &{ - }\frac{\eta \widehat{t} \sigma _{2l} Y_{l} T'}{T} - \frac{b_{2l} \sigma _{2l}^{2} Y_{l}^{2}}{T} - \frac{\mu _{l} \sigma _{1l} \sigma _{2l} X_{l} Y_{l}}{T} = -\eta \widehat{t} \sigma _{2l} Y_{l}'+ b_{0l} T + c_{l} \sigma _{2l} Y_{l} + \gamma _{l} \sigma _{2r} Y_{r}. \end{aligned}$$

Note that all the terms on the right-hand side of the equations (53)–(54) have order *n*; numerators on the left-hand side of the equations (53)–(54) have order 2*n*; denominators on the left-hand side of the equations (53)–(54) have order *n*. Thus, in order for the equations (53)–(54) to hold, the denominators must be canceled out, i.e., the following conditions must be satisfied:

55 $$\begin{aligned} & \eta \widehat{t} \sigma _{1l} X_{l}' - a_{0l} T = \sigma _{1l} \alpha _{l} X_{l}, \end{aligned}$$

56 $$\begin{aligned} & {-} \eta \widehat{t} \sigma _{2l} T'- b_{2l} \sigma _{2l}^{2} Y_{l} - \mu _{l} \sigma _{1l} \sigma _{2l} X_{l} = \sigma _{2l} \beta _{l} T, \end{aligned}$$

where $\alpha _{1}, \alpha _{2}, \beta _{1}, \beta _{2} \in \mathbb{R} \backslash \{ 0 \}$ are arbitrary constants.

Consequently, conditions (55)–(56) are necessary for the existence of solitary solutions (46) in the system (15)–(18). Note that conditions (55)–(56) can be rearranged as follows:

57 $$\begin{aligned} & a_{0l} T + \alpha _{l} \sigma _{1l} X_{l} - \eta \widehat{t} \sigma _{1l} X_{l}' = 0, \end{aligned}$$

58 $$\begin{aligned} & \eta \widehat{t} T' + \beta _{l} T + \mu _{l} \sigma _{1l} X_{l} + b_{2l} \sigma _{2l} Y_{l} = 0, \end{aligned}$$

where $l = 1,2$.

Inserting (55)–(56) into (53)–(54) yields the following system of algebraic equations:

59 $$\begin{aligned} & \alpha _{l} T = \eta \widehat{t} T' + a_{1l} T + a_{2l} \sigma _{1l} X_{l} + \lambda _{l} \sigma _{2l} Y_{l}, \end{aligned}$$

60 $$\begin{aligned} & \sigma _{2l} \beta _{l} Y_{l} = -\eta \widehat{t} \sigma _{2l} Y_{l}'+ b_{0l} T + c_{l} \sigma _{2l} Y_{l} + \gamma _{l} \sigma _{2r} Y_{r}, \end{aligned}$$

where $l,r = 1,2$; $l \neq r$.

Equations (59)–(60) correspond to the sufficient conditions for the existence of solitary solutions (46) in the system (15)–(18). Note that those conditions can be rearranged as follows:

61 $$\begin{aligned} & \eta \widehat{t} T' + (a_{1l} - \alpha _{l}) T + a_{2l} \sigma _{1l} X_{l} + \lambda _{l} \sigma _{2l} Y_{l} = 0, \end{aligned}$$

62 $$\begin{aligned} &\gamma _{l} \sigma _{2r} Y_{r} + (c_{l} - \beta _{l}) \sigma _{2l} Y_{l} + b_{0l} T - \eta \sigma _{2l} \widehat{t} Y_{l}' = 0. \end{aligned}$$

### Lemma 1

*Solitary solutions* (46) *satisfy the system* (15)*–*(18) *if and only if the conditions* (57)*–*(58) *and* (61)*–*(62) *hold true*.

## Determination of the maximal order of the solitary solution (46) to (15)–(18)

In this subsection the inverse balancing technique (see Sect. 3.3) is applied in order to express the coefficients of the system (15)–(18) in terms of the solitary solution (46) parameters, as well as to determine the maximal order of the solitary solution (46) to (15)–(18). Consider the following one-to-one mappings:

63 $$\begin{aligned} & T \mapsto \overrightarrow{T} = (1, \mbox{\ae }_{n-1}, \dots , \mbox{ \ae }_{0} ), \end{aligned}$$

64 $$\begin{aligned} & X_{l} \mapsto \overrightarrow{X_{l}} = (1, \chi _{(n-1)l}, \dots , \chi _{0l} ), \end{aligned}$$

65 $$\begin{aligned} & Y_{l} \mapsto \overrightarrow{Y_{l}} = (1, \theta _{(n-1)l}, \dots , \theta _{0l} ), \end{aligned}$$

66 $$\begin{aligned} & \widehat{t} T' \mapsto \overrightarrow{ \bigl(\widehat{t} T' \bigr)} = \bigl(n, (n-1) \mbox{\ae }_{n-1}, \dots , \mbox{\ae }_{1}, 0 \bigr), \end{aligned}$$

67 $$\begin{aligned} & \widehat{t} X_{l}' \mapsto \overrightarrow{ \bigl( \widehat{t} T' \bigr)} = \bigl(n, (n-1)\chi _{(n-1)l}, \dots , \chi _{1l},0 \bigr), \end{aligned}$$

68 $$\begin{aligned} & \widehat{t} Y_{l}' \mapsto \overrightarrow{ \bigl( \widehat{t} T' \bigr)} = \bigl(n, (n-1) \theta _{(n-1)l}, \dots , \theta _{1l},0 \bigr), \end{aligned}$$

where $l = 1,2$ and $X_{l}$, $Y_{l}$, *T* are defined in (47)–(49), respectively. Let $A_{1l}$, $A_{2l}$, $L_{1l}$, $L_{2l}$, $L_{3l}$, $N_{1l}$, $N_{2l}$, $N_{3l}$, $K_{1l}$, $K_{2l}$, $K_{3l} \in \mathbb{R}$; $l = 1,2$. Then using (63)–(68), necessary and sufficient conditions (57)–(58) and (61)–(62) can be rewritten in the vector form as follows:

69 $$\begin{aligned} & \overrightarrow{T} + A_{1l} \overrightarrow{X_{l}} + A_{2l} \overrightarrow{ \bigl(\widehat{t} X_{l}' \bigr)} = 0, \end{aligned}$$

70 $$\begin{aligned} & \overrightarrow{ \bigl(\widehat{t} T' \bigr)} + L_{1l} \overrightarrow{T} + L_{2l} \overrightarrow{X_{l}} + L_{3l} \overrightarrow{Y_{l}} = 0, \end{aligned}$$

71 $$\begin{aligned} & \overrightarrow{ \bigl(\widehat{t} T' \bigr)} + N_{1l} \overrightarrow{T} + N_{2l} \overrightarrow{X_{l}} + N_{3l} \overrightarrow{Y_{l}} = 0, \end{aligned}$$

72 $$\begin{aligned} & \overrightarrow{Y_{r}} + K_{1l} \overrightarrow{Y_{l}} + K_{2l} \overrightarrow{T} + K_{3l} \overrightarrow{ \bigl( \widehat{t} Y_{l}' \bigr)} = 0, \end{aligned}$$

where

73 $$\begin{aligned} & A_{1l} = \frac{\alpha _{l} \sigma _{1l}}{a_{0l}}, \qquad A_{2l} = - \frac{\eta \sigma _{1l}}{a_{0l}}, \end{aligned}$$

74 $$\begin{aligned} & L_{1l} = \frac{\beta _{l}}{\eta }, \qquad L_{2l} = \frac{\mu _{l} \sigma _{1l}}{\eta }, \qquad L_{3l} = \frac{b_{2l} \sigma _{2l}}{\eta }, \end{aligned}$$

75 $$\begin{aligned} & N_{1l} = \frac{a_{1l}-\alpha _{l}}{\eta }, \qquad N_{2l} = \frac{a_{2l} \sigma _{1l}}{\eta }, \qquad N_{3l} = \frac{\lambda _{l} \sigma _{2l}}{\eta }, \end{aligned}$$

76 $$\begin{aligned} & K_{1l} = \frac{(c_{l} - \beta _{l}) \sigma _{2l}}{\gamma _{l} \sigma _{2r}}, \qquad K_{2l} = \frac{b_{0l}}{\gamma _{l} \sigma _{2r}}, \qquad K_{3l} = -\frac{\eta \sigma _{2l}}{\gamma _{l} \sigma _{2r}}, \end{aligned}$$

for $l,r = 1,2$, $l \neq r$.

This section will consider three types of parameters:

- Parameters of the system of differential equations (15)–(18), namely $a_{kl}$, $b_{kl}$, $\lambda _{l}$, $\mu _{l}$, and $\gamma _{l}$, where $k = 0,1,2$; $l = 1,2$.
- Parameters of the solitary solution (46), namely *η*, $t_{0}$, $\sigma _{kl}$, $\mbox{\ae }_{m}$, $\chi _{ml}$, and $\theta _{ml}$, where $m = 0,\dots ,n-1$; $l,k = 1,2$.
- Auxiliary parameters, namely $\alpha _{l}$, $\beta _{l}$, $A_{kl}$, $L_{ml}$, $N_{ml}$, and $K_{ml}$, where $m = 1,2,3$; $l,k = 1,2$. As mentioned previously, parameters $\alpha _{l}$ and $\beta _{l}$ are required for the derivation of necessary and sufficient conditions (69)–(72) (see Eqs. (55)–(56)). Parameters $A_{kl}$, $L_{ml}$, $N_{ml}$, and $K_{ml}$ relate parameters of the system (15)–(18) to solitary solution parameters through necessary and sufficient conditions (69)–(72).

Note that if auxiliary and solitary solution (46) parameters could be determined by solving (69)–(72), then parameters of the system (15)–(18) could be expressed as follows:

77 $$\begin{aligned} & \begin{aligned} &a_{0l} = -\eta \frac{\sigma _{1l}}{A_{2l}}, \qquad a_{1l} = \eta \biggl(N_{1l} - \frac{A_{1l}}{A_{2l}} \biggr), \qquad a_{2l} = \frac{N_{2l} \eta }{\sigma _{1l}}, \\ &\lambda _{l} = \frac{N_{3l} \eta }{\sigma _{2l}}, \qquad b_{0l} = - \frac{\eta \sigma _{2l} K_{2l}}{K_{3l}}, \end{aligned} \end{aligned}$$

78 $$\begin{aligned} & b_{1l} = -\eta \biggl(\frac{K_{1l}}{K_{3l}} + \frac{\sigma _{2l}}{K_{3l} \sigma _{2r}} + L_{1l} \biggr), \qquad b_{2l} = \frac{L_{3l} \eta }{\sigma _{2l}}, \qquad \mu _{l} = \frac{L_{2l} \eta }{\sigma _{1l}}, \qquad \gamma _{l} = - \frac{\eta \sigma _{2l}}{K_{3l} \sigma _{2r}}, \end{aligned}$$

where $l, r = 1,2$, $l \neq r$.

Insert (63), (64), and (67) into (69) and consider the elements of the obtained vector results in the following system of equations with respect to $A_{1l}$, $A_{2l}$, $\mbox{\ae }_{k}$, and $\chi _{kl}$ (where $k=0,\dots ,n-1$, $l = 1,2$):

79 $$ \textstyle\begin{cases} 1 + A_{1l} + n A_{2l} = 0, \\ \mbox{\ae }_{k} + A_{1l} \chi _{kl} + A_{2l} k \chi _{kl} = 0, \quad k = n-1, \dots , 0, \end{cases} $$

where $l = 1,2$. Note that system (79) has $2n+2$ equations and $3n+4$ unknowns. Solving (79) yields:

80 $$ A_{2l} = -\frac{1+A_{1l}}{n}, \qquad \chi _{kl} = \frac{\mbox{\ae }_{k}}{1+(n-k)A_{2l}} = p_{kl} \mbox{\ae }_{k}, \quad A_{1l}, \mbox{\ae }_{k} \in \mathbb{R}, $$

where $p_{kl} = \frac{1}{1+(n-k)A_{2l}}$ and $k=0,\dots ,n-1$, $l = 1,2$.

Inserting (63)–(66) into (70) results in the following system of linear equations with respect to $L_{1l}$, $L_{2l}$, $L_{3l}$:

81 $$ \textstyle\begin{cases} n + L_{1l} + L_{2l} + L_{3l} = 0, \\ k \mbox{\ae }_{k} + L_{1l} \mbox{\ae }_{k} + L_{2l} \chi _{kl} + L_{3l} \theta _{kl} = 0, \quad k = n-1, \dots , 0, \end{cases} $$

where $l = 1,2$.

Let

82 $$ \theta _{kl} = h_{kl} \mbox{\ae }_{k}, \quad h_{kl} \in \mathbb{C}, $$

where $k=0,\dots ,n-1$, $l = 1,2$.

Then dividing the second equation in (81) by $\mbox{\ae }_{k}$ yields

83 $$ \textstyle\begin{cases} n + L_{1l} + L_{2l} + L_{3l} = 0, \\ k + L_{1l} + L_{2l} p_{kl} + L_{3l} h_{kl} = 0, \quad k = n-1, \dots , 0, \end{cases} $$

where $l = 1,2$. Note that if $\mbox{\ae }_{k} = 0$ for some $k=0,\dots ,n-1$, then the respective equation always holds true. Analogously, inserting (63)–(66) into (71) results in the following system with respect to $N_{1l}$, $N_{2l}$, $N_{3l}$:

84 $$ \textstyle\begin{cases} n + N_{1l} + N_{2l} + N_{3l} = 0, \\ k + N_{1l} + N_{2l} p_{kl} + N_{3l} h_{kl} = 0, \quad k = n-1, \dots , 0, \end{cases} $$

where $l = 1,2$.

Note that systems (81) and (84) are identical. When (46) are kink solitary solutions ($n=1$), systems (81) and (84) have infinitely many solutions of the following form:

85 $$\begin{aligned} &L_{1l} = \frac{(1 + L_{3l}) p_{0l} - L_{3l} h_{0l}}{1 - p_{0l}}, \qquad L_{2l} = \frac{-(1 + L_{3l}) + L_{3l} h_{0l}}{1 - p_{0l}}, \quad L_{3l}\in \mathbb{R}, \end{aligned}$$

86 $$\begin{aligned} &N_{1l} = \frac{(1 + N_{3l}) p_{0l} - N_{3l} h_{0l}}{1 - p_{0l}}, \qquad N_{2l} = \frac{-(1 + N_{3l}) + N_{3l} h_{0l}}{1 - p_{0l}}, \quad N_{3l}\in \mathbb{R}, \end{aligned}$$

where $l = 1,2$. Note that in this case solutions $L_{kl}$ and $N_{kl}$ ($k=1,2,3$) are not necessarily equal.

However, when $n \ge 2$, systems (81) and (84) can only have a single unique solution:

87 $$ \begin{aligned} &L_{1l} = N_{1l} = \frac{(p_{1l} n - 1) h_{0l} - p_{0l} (n h_{1l} - 1)}{(1 - p_{1l}) h_{0l} + ( h_{1l} -1) p_{0l} + (p_{1l} - h_{1l})}, \\ &L_{2l} = N_{2l} = \frac{(1 - n) h_{0l} - 1 + n h_{1l}}{(1 - p_{1l}) h_{0l} + ( h_{1l} -1) p_{0l} + (p_{1l} - h_{1l})}, \\ &L_{3l} = N_{2l} = \frac{(n - 1) p_{0l} + 1 - n p_{1l}}{(1 - p_{1l}) h_{0l} + (h_{1l} -1) p_{0l} + (p_{1l} - h_{1l})} \end{aligned} $$

if and only if the following conditions with respect to solitary solution (46) parameters hold true:

88 $$\begin{aligned} &k + L_{1l} + L_{2l} p_{kl} + L_{3l} h_{kl} = 0, \quad k = 2, \dots , n-1, \end{aligned}$$

89 $$\begin{aligned} & (1 - p_{1l}) h_{0l} + (-1 + h_{1l}) p_{0l} + p_{1l} - h_{1l} \ne 0, \end{aligned}$$

where $l = 1,2$.

Note that in this case, applying $L_{kl} = N_{kl}$ ($k=1,2,3$) to (73)–(76) yields

90 $$ a_{2l} = \mu _{l}, \qquad b_{2l} = \lambda _{l}, $$

where $l= 1,2$.

Applying (63), (65), and (68) to (72) results in the following system of linear equations with respect to $K_{1l}$, $K_{2l}$, and $K_{3l}$:

91 $$ \textstyle\begin{cases} 1 + K_{1l} + K_{2l} + n K_{3l} = 0, \\ h_{kr} + K_{1l} h_{kl} + K_{2l} + K_{3l} k h_{kl} = 0, \quad k = n-1, \dots , 0, \end{cases} $$

where $l, r = 1,2$, $l \neq r$.

The system (91) yields the expressions for $K_{1l}$, $K_{2l}$:

92 $$ K_{1l} = \frac{h_{0r}-n K_{3l}-1}{1 - h_{0l}}, \qquad K_{2l} = \frac{(n K_{3l} + 1) h_{0l} - h_{0r}}{1 - h_{0l}}, $$

and the following conditions:

93 $$\begin{aligned} & h_{kr} + K_{1l} h_{kl} + K_{2l} + K_{3l} k h_{kl} = 0, \quad k = 1, \dots , n-1, \end{aligned}$$

where $l, r = 1,2$, $l \neq r$.

Thus, necessary and sufficient conditions for the existence of solitary solutions (46) to the system (15)–(18) can be reformulated in terms of solitary solution parameters as follows:

### Lemma 2

*Solitary solutions* (46) *satisfy the system* (15)*–*(18) *if and only if the conditions* (88) *and* (93) *hold true*.

Note that applying (80) and (82) to (46) yields the following expression of the solitary solution:

94 $$\begin{aligned} & x_{l} (t ) = \widehat{x_{l}} (\widehat{t} ) = \sigma _{1l} \frac{X_{l}}{T} = \sigma _{1l} \frac{ (\widehat{t} )^{n} + p_{(n-1)l} \mbox{\ae }_{n-1} (\widehat{t} )^{n-1} + \cdots + p_{0l} \mbox{\ae }_{0}}{ (\widehat{t} )^{n} + \mbox{\ae }_{n-1} (\widehat{t} )^{n-1} + \cdots + \mbox{\ae }_{0}}, \end{aligned}$$

95 $$\begin{aligned} & y_{l} (t ) = \widehat{y_{l}} (\widehat{t} ) = \sigma _{2l} \frac{Y_{l}}{T} = \sigma _{2l} \frac{ (\widehat{t} )^{n} + h_{(n-1)l} \mbox{\ae }_{n-1} (\widehat{t} )^{n-1} + \cdots + h_{0l} \mbox{\ae }_{0}}{ (\widehat{t} )^{n} + \mbox{\ae }_{n-1} (\widehat{t} )^{n-1} + \cdots + \mbox{\ae }_{0}}, \end{aligned}$$

for $l = 1,2$.

Algebraically solving the system of necessary and sufficient conditions defined in Lemma 2 for various values of *n* yields the conclusion, summarized in the lemma below.

### Lemma 3

*The system of differential equations* (15)*–*(18) *can admit solitary solutions of any order* $n \in \mathbb{N}$. *However*, *two cases are present*:

- *If* $n \le 3$, *the system of necessary and sufficient conditions defined in Lemma* 2*can be solved without additional constraints on solitary solution parameters*. *Moreover*, *selecting different values of* $\mbox{\ae }_{k}$, $k = 0, \dots , n-1$ *in* (94)*–*(95) *generates an infinite number of solitary solutions corresponding to a single system of differential equations* (15)*–*(18). *Note that in the case of* $n = 2, 3$, *constraints* (90) *on differential equation parameters must be satisfied in order to ensure the existence of the solitary solution*, *whereas for* $n = 1$ *these constraints are unnecessary*.
- *If* $n > 3$, *the system of necessary and sufficient conditions defined in Lemma* 2*can be solved if any* $(n-3)$ *parameters* $\mbox{\ae }_{k}$, $k \in \{0, \dots , n-1\}$ *are equal to zero*. *Then*, *selecting different values of the remaining parameters* $\mbox{\ae }_{k}$ *in* (94)*–*(95) *generates an infinite number of solitary solutions corresponding to a single system of differential equations* (15)*–*(18). *Moreover*, *constraints* (90) *on the differential equation parameters must be satisfied in order to ensure the existence of the solitary solution*.

The auxiliary parameters $A_{kl}$, $L_{ml}$, $N_{ml}$, and $K_{ml}$ form an essential link between the parameters of the system (15)–(18) and the solitary solution (46). If it is possible to determine the auxiliary parameters from the solitary solution, the system parameters can be computed via (77) and (78). Conversely, if the system parameters are known they can be used to determine auxiliary parameters, which in turn yield the solitary solution parameters via (69)–(76).

## Computational experiments. Third-order solitary solutions to (15)–(18)

### Analytical computation of auxiliary parameters from solitary solution parameters

In this subsection, the presented derivations are illustrated: starting from the given third-order solitary solutions ($n=3$), the auxiliary parameters are first derived. From the auxiliary parameters, the coefficients of the system (15)–(18) are then computed.

The following example demonstrates these steps in detail, closely following the derivations presented in Sects. 4 and 5. See Sect. 6.2 for a more application-oriented example, where solitary solutions to a given system of diffusively coupled Lotka–Volterra equations are constructed.

Lemma 2 is applied in order to show that system (15)–(18) can admit third order ($n=3$) solitary solutions:

96 $$\begin{aligned} & x_{1} (t ) = \widehat{x_{1}} (\widehat{t} ) = \sigma _{11} \frac{X_{1}}{T} = \sigma _{11} \frac{ (\widehat{t} )^{3} + p_{21} \mbox{\ae }_{2} (\widehat{t} )^{2} + p_{11} \mbox{\ae }_{1} \widehat{t} + p_{01} \mbox{\ae }_{0}}{ (\widehat{t} )^{3} + \mbox{\ae }_{2} (\widehat{t} )^{2} + \mbox{\ae }_{1} \widehat{t} + \mbox{\ae }_{0}}, \end{aligned}$$

97 $$\begin{aligned} & x_{2} (t ) = \widehat{x_{2}} (\widehat{t} ) = \sigma _{12} \frac{X_{2}}{T} = \sigma _{12} \frac{ (\widehat{t} )^{3} + p_{22} \mbox{\ae }_{2} (\widehat{t} )^{2} + p_{12} \mbox{\ae }_{1} \widehat{t} + p_{02} \mbox{\ae }_{0}}{ (\widehat{t} )^{3} + \mbox{\ae }_{2} (\widehat{t} )^{2} + \mbox{\ae }_{1} \widehat{t} + \mbox{\ae }_{0}}, \end{aligned}$$

98 $$\begin{aligned} & y_{1} (t ) = \widehat{y_{1}} (\widehat{t} ) = \sigma _{21} \frac{Y_{1}}{T} = \sigma _{21} \frac{ (\widehat{t} )^{3} + h_{21} \mbox{\ae }_{2} (\widehat{t} )^{2} + h_{11} \mbox{\ae }_{1} \widehat{t} + h_{01} \mbox{\ae }_{0}}{ (\widehat{t} )^{3} + \mbox{\ae }_{2} (\widehat{t} )^{2} + \mbox{\ae }_{1} \widehat{t} + \mbox{\ae }_{0}}, \end{aligned}$$

99 $$\begin{aligned} & y_{2} (t ) = \widehat{y_{2}} (\widehat{t} ) = \sigma _{22} \frac{Y_{2}}{T} = \sigma _{22} \frac{ (\widehat{t} )^{3} + h_{22} \mbox{\ae }_{2} (\widehat{t} )^{2} + h_{12} \mbox{\ae }_{1} \widehat{t} + h_{02} \mbox{\ae }_{0}}{ (\widehat{t} )^{3} + \mbox{\ae }_{2} (\widehat{t} )^{2} + \mbox{\ae }_{1} \widehat{t} + \mbox{\ae }_{0}}. \end{aligned}$$

As shown in Sect. 5, solitary solutions (96)–(99) satisfy the model (15)–(18) if and only if the following conditions hold true:

100 $$\begin{aligned} &2 + L_{1l} + L_{2l} p_{2l} + L_{3l} h_{2l} = 0, \end{aligned}$$

101 $$\begin{aligned} & h_{1r} + K_{1l} h_{1l} + K_{2l} + K_{3l} h_{1l} = 0, \end{aligned}$$

102 $$\begin{aligned} & h_{2r} + K_{1l} h_{2l} + K_{2l} + 2 K_{3l} h_{2l} = 0, \end{aligned}$$

where

103 $$\begin{aligned} &L_{1l} = \frac{(3 p_{1l} - 1) h_{0l} - p_{0l} (3 h_{1l} - 1)}{(1 - p_{1l}) h_{0l} + ( h_{1l} -1) p_{0l} + (p_{1l} - h_{1l})}, \end{aligned}$$

104 $$\begin{aligned} &L_{2l} = \frac{-2 h_{0l} - 1 + 3 h_{1l}}{(1 - p_{1l}) h_{0l} + ( h_{1l} -1) p_{0l} + (p_{1l} - h_{1l})}, \end{aligned}$$

105 $$\begin{aligned} &L_{3l} = \frac{2 p_{0l} + 1 - 3 p_{1l}}{(1 - p_{1l}) h_{0l} + (h_{1l} -1) p_{0l} + (p_{1l} - h_{1l})}, \end{aligned}$$

106 $$\begin{aligned} &K_{1l} = \frac{h_{0r}- 3 K_{3l}-1}{1 - h_{0l}}, \end{aligned}$$

107 $$\begin{aligned} &K_{2l} = \frac{(3 K_{3l} + 1) h_{0l} - h_{0r}}{1 - h_{0l}}, \end{aligned}$$

108 $$\begin{aligned} &p_{kl} = \frac{1}{1+(3-k)A_{2l}}, \end{aligned}$$

109 $$\begin{aligned} &A_{2l} = -\frac{1+A_{1l}}{3}, \end{aligned}$$

for $l,r = 1,2$; $l \neq r$, and $k=0,1,2$. Note that the system (100)–(102) has 6 equations and 10 unknowns, namely, $K_{31}$, $K_{32}$, $A_{11}$, $A_{12}$, $h_{01}$, $h_{02}$, $h_{11}$, $h_{12}$, $h_{21}$, and $h_{22}$. Four unknowns from the system (100)–(102) can be chosen arbitrarily, however, they can be used to determine if the solitary solutions have the desired number of maxima and minima. In this example, the following parameter values are selected to ensure that at least two solitary solutions have no fewer than three extrema:

110 $$ A_{11} = \frac{4}{5}, \qquad A_{12} = \frac{2}{5}, \qquad h_{12} = \frac{139\mbox{,}919}{7619}, \qquad h_{22} = \frac{39\mbox{,}493}{15\mbox{,}238}. $$

Then, solving (100)–(102) with respect to $K_{31}$, $K_{32}$, $h_{01}$, $h_{02}$, $h_{11}$, $h_{21}$ yields:

111 $$\begin{aligned} \begin{aligned} &K_{31} = - \frac{7\mbox{,}834\mbox{,}855}{274\mbox{,}284}, \qquad K_{32} = - \frac{685\mbox{,}710}{223\mbox{,}853}, \qquad h_{01} = -\frac{34\mbox{,}171}{31\mbox{,}979}, \\ & h_{02} = -\frac{10\mbox{,}021}{7619}, \qquad h_{11} = - \frac{170\mbox{,}881}{31\mbox{,}979}, \qquad h_{21} = \frac{89\mbox{,}309}{31\mbox{,}979}. \end{aligned} \end{aligned}$$

Using (110) and (111), parameters (103)–(109) can be evaluated as follows:

112 $$\begin{aligned} \begin{aligned} &L_{11} = - \frac{9929}{4410}, \qquad L_{12} = -\frac{12\mbox{,}029}{4410}, \qquad L_{21} = -8, \qquad L_{22} = -2, \\ &L_{31} = \frac{31\mbox{,}979}{4410}, \qquad L_{32} = \frac{7619}{4410}, \qquad K_{11} = \frac{199\mbox{,}005\mbox{,}317}{4\mbox{,}937\mbox{,}112}, \qquad K_{12}= \frac{33\mbox{,}736\mbox{,}932}{10\mbox{,}968\mbox{,}797}, \\ &K_{21} = \frac{219\mbox{,}139\mbox{,}741}{4\mbox{,}937\mbox{,}112}, \qquad K_{22} = \frac{56\mbox{,}093\mbox{,}641}{10\mbox{,}968\mbox{,}797}, \qquad p_{01} = -\frac{5}{4}, \qquad p_{02} = -\frac{5}{2}, \\ &p_{11} = -5, \qquad p_{12} = 15, \qquad p_{21} = \frac{5}{2}, \qquad p_{22} = \frac{15}{8}, \\ &A_{21} = -\frac{3}{5}, \qquad A_{22} = - \frac{7}{15}. \end{aligned} \end{aligned}$$

Since parameters (110) and (111) ensure the validity of conditions (100)–(102), solitary solution (96)–(97) parameters æ_0_, æ_1_, æ_2_, $\sigma _{11}$, $\sigma _{12}$, $\sigma _{21}$, $\sigma _{22}$, *η*, $t_{0}$ can be chosen freely. Moreover, selecting different values of æ_0_, æ_1_, æ_2_ generates an infinite number of third-order solitary solutions corresponding to a single system of differential equations (15)–(18). Consider the case with

113 $$\begin{aligned} \begin{aligned} &\mbox{\ae }_{0} = 8, \qquad \mbox{\ae }_{1} = 14, \qquad \mbox{\ae }_{2} = 7, \qquad \eta = 4, \qquad t_{0} = -5, \\ &\sigma _{11} = \frac{3}{5}, \qquad \sigma _{12} = \frac{7}{15}, \qquad \sigma _{21} = \frac{7\mbox{,}834\mbox{,}855}{274\mbox{,}284}, \qquad \sigma _{22} = \frac{685\mbox{,}710}{223\mbox{,}853}. \end{aligned} \end{aligned}$$

Then, inserting obtained parameter values and applying (20) to solitons (96)–(97) yields:

114 $$\begin{aligned} & x_{1} (t ) = \frac{3 (2 \exp {(12t-15)} + 35 \exp {(8t-10)} - 140 \exp {(4t-5)} - 20 )}{10 (\exp {(12t-15)} + 7 \exp {(8t-10)} + 14 \exp {(4t-5)} + 8 )}, \end{aligned}$$

115 $$\begin{aligned} & x_{2} (t ) = \frac{7 (8 \exp {(12t-15)} + 105 \exp {(8t-10)} + 1680 \exp {(4t-5)} - 160 )}{120 (\exp {(12t-15)} + 7 \exp {(8t-10)} + 14 \exp {(4t-5)} + 8 )}, \end{aligned}$$

116 $$\begin{aligned} & \begin{aligned}[b] y_{1} (t ) &= \bigl(245 (31\mbox{,}979 \exp {(12t-15)} + 625\mbox{,}163 \exp {(8t-10)}\\ &\quad {} - 2\mbox{,}392\mbox{,}334 \exp {(4t-5)} - 273\mbox{,}368 )\bigr)\\ &\quad {}/\bigl(274\mbox{,}284 (\exp {(12t-15)} + 7 \exp {(8t-10)} + 14 \exp {(4t-5)} + 8 )\bigr), \end{aligned} \end{aligned}$$

117 $$\begin{aligned} & \begin{aligned}[b] y_{2} (t ) &= \bigl(45 (15\mbox{,}238 \exp {(12t-15)} + 276\mbox{,}451 \exp {(8t-10)} \\ &\quad {}+ 3\mbox{,}917\mbox{,}732 \exp {(4t-5)} - 160\mbox{,}336 )\bigr)\\ &\quad {}/\bigl(223\mbox{,}853 (\exp {(12t-15)} + 7 \exp {(8t-10)} + 14 \exp {(4t-5)} + 8 )\bigr). \end{aligned} \end{aligned}$$

Once the auxiliary and solitary solution parameters are computed, (77)–(78) can be used to obtain the parameters of the system (15)–(18) which admits the solitary solution (114)–(117). The system (15)–(18) reads:

118 $$\begin{aligned} &x_{1}' = 1 - \frac{4049}{4410} x_{1} - \frac{40}{3} x_{1}^{2} + \frac{15\mbox{,}238}{60\mbox{,}025} x_{1} y_{1}, \end{aligned}$$

119 $$\begin{aligned} &y_{1}' = \frac{219\mbox{,}139\mbox{,}741}{4\mbox{,}937\mbox{,}112} - \frac{5\mbox{,}755\mbox{,}739}{11\mbox{,}199\mbox{,}930} y_{1} + \frac{15\mbox{,}238}{60\mbox{,}025} y_{1}^{2} - \frac{40}{3} x_{1} y_{1} + \frac{223\mbox{,}853}{685\mbox{,}710} (y_{2} - y_{1} ), \end{aligned}$$

120 $$\begin{aligned} &x_{2}' = 1 - \frac{8249}{4410} x_{2} - \frac{30}{7} x_{2}^{2} + \frac{31\mbox{,}979}{56\mbox{,}700} x_{2} y_{2}, \end{aligned}$$

121 $$\begin{aligned} &y_{2}' = \frac{56\mbox{,}093\mbox{,}641}{10\mbox{,}968\mbox{,}797} - \frac{238\mbox{,}135\mbox{,}267}{141\mbox{,}027\mbox{,}390} y_{2} + \frac{31\mbox{,}979}{56\mbox{,}700} y_{2}^{2} - \frac{30}{7} x_{2} y_{2} + \frac{274\mbox{,}284}{7\mbox{,}834\mbox{,}855} (y_{1} - y_{2} ). \end{aligned}$$

### Construction of solitary solutions to a diffusively coupled Lotka–Volterra system

Let us consider the following system of diffusively coupled Lotka–Volterra equations:

122 $$\begin{aligned} &{x_{1}' = \frac{5}{3} - \frac{16}{9} x_{1} - \frac{68}{5} x_{1}^{2} + \frac{617}{135} x_{1} y_{1},} \end{aligned}$$

123 $$\begin{aligned} &{y_{1}' = \frac{153\mbox{,}745}{31\mbox{,}467} - \frac{656\mbox{,}207}{377\mbox{,}604} y_{1} + \frac{617}{135} y_{1}^{2} - \frac{68}{5} x_{1} y_{1} - \frac{243}{41\mbox{,}956} (y_{2} - y_{1} ),} \end{aligned}$$

124 $$\begin{aligned} &{x_{2}' = \frac{30}{7} - \frac{172}{63} x_{2} + \frac{68}{315} x_{2}^{2} + \frac{7}{180} x_{2} y_{2},} \end{aligned}$$

125 $$\begin{aligned} &{y_{2}' = -\frac{740}{153} + \frac{6317}{3213} y_{2} + \frac{7}{180} y_{2}^{2} + \frac{68}{315} x_{2} y_{2} + \frac{2468}{459} (y_{1} - y_{2} ).} \end{aligned}$$

Third-order solitary solutions of the form (96)–(99) to the above system are constructed in this subsection via the computational scheme presented in the paper.

Using the parameters of the system of differential equations (122)–(125), auxiliary parameters $\alpha _{l}$, $\beta _{l}$, $A_{kl}$, $L_{ml}$, $N_{ml}$, and $K_{ml}$ ($m = 1,2,3$; $l,k = 1,2$), as well as solitary solutions parameters *η*, $t_{0}$, and $\sigma _{kl}$ ($l,k = 1,2$), can be obtained by solving (73)–(76). One of the possible sets of such parameters (solutions of (73)–(76)) reads:

126 $$\begin{aligned} \begin{aligned} &{\alpha _{1} = \frac{4}{3}, \qquad \alpha _{2} = \frac{6}{7}, \qquad \beta _{1} = -\frac{28}{9}, \qquad \beta _{2} = - \frac{226}{63},\qquad A_{11} = \frac{4}{5}, \qquad A_{12} = \frac{2}{5},} \\ &{A_{21} = -\frac{3}{5}, \qquad A_{22} = - \frac{7}{15}, \qquad L_{11} = N_{11} = - \frac{28}{9}, \qquad L_{12} = N_{12} = - \frac{226}{63},} \\ &{L_{21} = N_{21} = -\frac{68}{5}, \qquad L_{22} = N_{22} = \frac{136}{315}, \qquad L_{31} = N_{31} = \frac{617}{45}, } \\ &{L_{32} = N_{32} = \frac{7}{45}, \qquad K_{11} = - \frac{130\mbox{,}187}{729}, \qquad K_{12}= \frac{27}{617},} \\ &{K_{21} = -\frac{153\mbox{,}745}{729}, \qquad K_{22} = - \frac{185}{617}, \qquad K_{31} = \frac{10\mbox{,}489}{81}, \qquad K_{31} = -\frac{153}{617},} \\ &{\eta = 1, \qquad t_{0} = -5, \qquad \sigma _{11} = 1, \qquad \sigma _{12} = 2, \qquad \sigma _{21} = 3, \qquad \sigma _{22} = 4.} \end{aligned} \end{aligned}$$

Using the obtained values (126), the rest of solitary solution (96)–(99) parameters ($\mbox{\ae }_{m}$, $p_{ml}$, and $h_{ml}$, $m = 0,1,2$; $l = 1,2$) can be derived by solving (80), (87), (88), (92), and (93):

127 $$\begin{aligned} \begin{aligned} &{\mbox{\ae }_{0} = 1, \qquad \mbox{\ae }_{1} = 5, \qquad \mbox{\ae }_{2} = 15,} \\ &{p_{01} = -\frac{5}{4}, \qquad p_{02} = - \frac{5}{2}, \qquad p_{11} = -5, \qquad p_{12} = 15,} \\ &{p_{21} = \frac{5}{2}, \qquad p_{22} = \frac{15}{8}, \qquad h_{01} = - \frac{625}{617}, \qquad h_{02} = 30,} \\ &{h_{11} = -\frac{2965}{617}, \qquad h_{12} = -25, \qquad h_{21} = \frac{1580}{617}, \qquad h_{22} = 5.} \end{aligned} \end{aligned}$$

Thus, third-order solitary solutions to the system (122)–(125) read:

128 $$\begin{aligned} &{x_{1} (t ) = \frac{4 \exp {(3t-15)} + 150 \exp {(2t-10)} - 100 \exp {(t-5)} - 5}{4 (\exp {(3t-15)} + 15 \exp {(2t-10)} + 5 \exp {(t-5)} + 1 )},} \end{aligned}$$

129 $$\begin{aligned} &{x_{2} (t ) = \frac{8 \exp {(3t-15)} + 225 \exp {(2t-10)} + 600 \exp {(t-5)} - 20}{4 (\exp {(3t-15)} + 15 \exp {(2t-10)} + 5 \exp {(t-5)} + 1 )},} \end{aligned}$$

130 $$\begin{aligned} &{y_{1} (t ) = \frac{3 (617 \exp {(3t-15)} + 23\mbox{,}700 \exp {(2t-10)} - 14\mbox{,}825 \exp {(t-5)} - 625 )}{617 (\exp {(3t-15)} + 15 \exp {(2t-10)} + 5 \exp {(t-5)} + 1 )},} \end{aligned}$$

131 $$\begin{aligned} &{y_{2} (t ) = \frac{4 (\exp {(3t-15)} + 75 \exp {(2t-10)} - 125 \exp {(t-5)} + 30 )}{\exp {(3t-15)} + 15 \exp {(2t-10)} + 5 \exp {(t-5)} + 1}.} \end{aligned}$$

**Figure 3:**
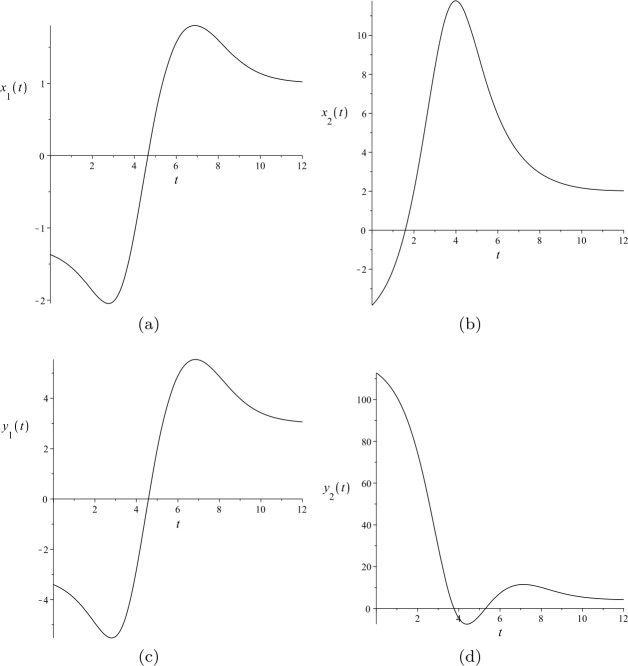
Third-order solitary solutions to (122)–(125): (**a**) $x_{1}(t)$; (**b**) $x_{2}(t)$; (**c**) $y_{1}(t)$; and (**d**) $y_{2}(t)$

The third-order solitary solutions (128)–(131) to (122)–(125) are illustrated in Fig. 3.

## Concluding remarks

It is well known that separatrices in the phase space play a pivotal role in understanding the evolution of solutions to nonlinear dynamical systems. As a rule of thumb, separatrices are usually represented by soliton-type solutions [25, 36]. A small impulse can be used to control the evolution of the transient trajectories of different nonlinear systems – provided it is possible to derive the analytic structure of separatrices in the phase space.

Kink solitary solutions do represent the separatrix between the silent mode and the firing mode of a dendritic neuron represented by a system of nonlinear differential equations [36]. Dark solitary solutions do represent a system of separatrices in the paradigmatic Hodgkin–Huxley model [36]. A control technique based on small impulses for silencing a random network of such neurons is proposed in [11, 36].

It was demonstrated in this paper that solitary solutions of an arbitrary order do exist in the diffusively coupled Lotka–Volterra systems. Necessary and sufficient conditions for the existence of such solutions were derived in terms of the system and solution parameters using the inverse balancing technique.

Finding soliton-type solutions to the meta-model of coupled Lotka–Volterra systems would allow us to identify the structure of separatrices in the 4-dimensional phase space. The knowledge of the system of separatrices would serve for designing algorithms for the control of transient processes. The meta-model of coupled Lotka–Volterra systems has some connections with the phenomenological model of metastasis and the SEIR COVID-19 model. Tuning these connections and designing algorithms for the control of transient processes remains a definite objective of future research.

## Data Availability

No data was used to obtain the results of this manuscript.
